# Silvol in Gonorrhea

**Published:** 1916-10

**Authors:** E. P. Merritt

**Affiliations:** Atlanta, Ga.; Instructor in Genito-Urinery Surgery, Atlanta Medical College; Medical Department of Emory University. Urologist to Atlanta Anti-Tuberculosis Association


### SILVOL IN GONORRHEA.

By E. P. Merritt, M. D., Atlanta, Ga.

Instructor in Genito-Urinary Surgery, Atlanta Medical College; Medical Department of Emory University. Urologist to Atlanta Anti-Tuberculosis Association.

With all the various silver preparations making their appearance upon the medical field it is really difficult to give them all a trial, but if one is worth while, it will in due time be discovered. My experience with Silvol is that I tried it as I would any other preparation. In the beginning I had no more faith in Silvol than I had in previous preparations I had already tried. I have used it in my practice for some time and my results have been very gratifying. My patients have felt no discomfort from its use. I soon discovered that it was one of the few to be chosen—in acute gonorrhea—for quick cures.

I have treated numbers of cases of acute gonorrhea with this preparation. I also find that chancroids will yield to it readily in some cases of mild infection. The question may be asked—why do I use Silvol? It seems to irritate the mucous-lining as little as any other preparation, and its germicidal effect is sure. It can easily be used in a 10 per cent. solution without the least discomfort to the patient. The following case histories or reports, taken at random from my files, will serve to illustrate my method of applying this splendidly meritorious preparation:

Case No. 1. Name: Miss B. Age: 21. Sex: Female. Came to me for treatment June 28th, 1916. She gave the following history, three or four days previous she noticed a burning and itching sensation in the vagina, aggravated by urination; also some discharge; she had never experienced anything like this before. On examination the meatus urinaris was red and swollen—by pressing forward a smear was taken, and gonococci was readily found; smear from the cervix was negative; the urine was cloudy with pus and shreds. Diagnosis: Acute gonorrhea. Treatment: Instillation of 10 per cent. Silvol solution in urethra and bladder twice daily for ten days, using potassium permanganate for vaginal douche once daily. After ten days' treatment patient was dismissed as cured. Several examinations have been made since she was dismissed and I find her to be absolutely well. I consider this a remarkably short time to cure a woman.

Case No. 2. Name: S. C. Age: 26. Sex: Male. Appeared for treatment August 1st, 1916. Duration of discharge only a few days he said. On examination I found slight discharge; smear made and examination revealed gonococci; urine, first glass, very cloudy; urine, second glass, very clear. Diagnosis: Acute gonorrhea. Treatment: Injection of a 10 per cent. solution Silvol three times daily; bicarbonate sodae internally. Treatment continued for five days and then left off, with urine clear and no discharge. Dismissed as cured. Examined again August 21st, and found to be absolutely well.

Case No. 3. Name: F. G. G. Age: 27. Sex: Male. Appeared for treatment July 15th, 1916. Said he noticed discharge on same date. On examination found discharge; smear made and on examination I found the gonococci present. Urine, first glass, very cloudy with pus; urine, second glass, clear. Diagnosis: Acute gonorrhea. Treatment: Injection of 10 per cent. solution Silvol four times daily for six days. July 21st, 1916, dismissed as cured. Has noticed no further trouble.

Conclusion: I could give any number of case histories, but in acute gonorrhea it would be the "same old story" told over and over again to repeat them. However, let it be understood that I have not cured every case of gonorrhea treated with Silvol or any other preparation, but my per cent. of cases cured with Silvol are above any other preparation I have used. This preparation will certainly equal any other silver preparation and personally, I like it better. Of course, if the treatment is not carried out right, the results will not be so gratifying. I think to get the best results from Silvol in the treatment of gonorrhea, the treatment should be carried out as follows: A solution of Silvol, 10 per cent. should be made up daily. Injections of same should be placed in urethra every time. patient urinates for the first four or five days. This would mean three or four injections daily as the average patient can hold his urine from three to six hours. Have patient grasp penis with thumb and index finger about three or four inches back, so as to keep solution from going back and also for dilating the anterior urethra folds. In most cases the urine should be alkaline, which may be accomplished with bicarbonate soda tid.

926 Candler Bldg.

